# Supplementary material for: Local Sustained GM-CSF Delivery by Genetically Engineered Encapsulated Cells Enhanced Both Cellular and Humoral SARS-CoV-2 Spike-Specific Immune Response in an Experimental Murine Spike DNA Vaccination Model
Source: Vaccines (Basel). 2021 May 10;9(5):484. doi: 10.3390/vaccines9050484 (PMC8151995; doi:10.3390/vaccines9050484)
Supplement: Supplementary file 1 [file vaccines-09-00484-s001.zip › vaccines-1204503-supplementary.pdf]

Supplementary Material

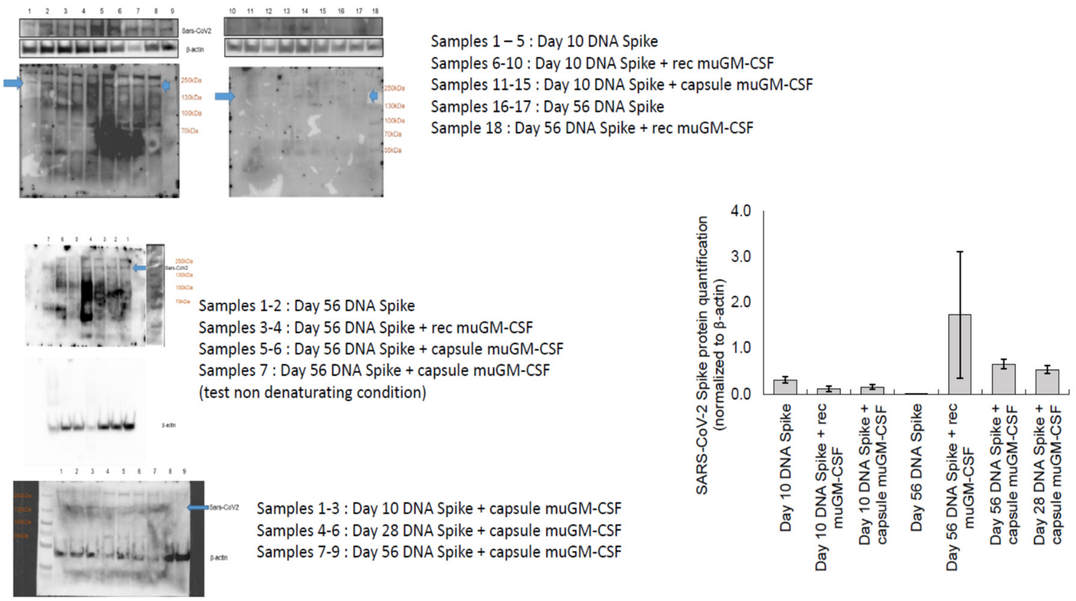

Figure S1. Uncropped Western Blot.

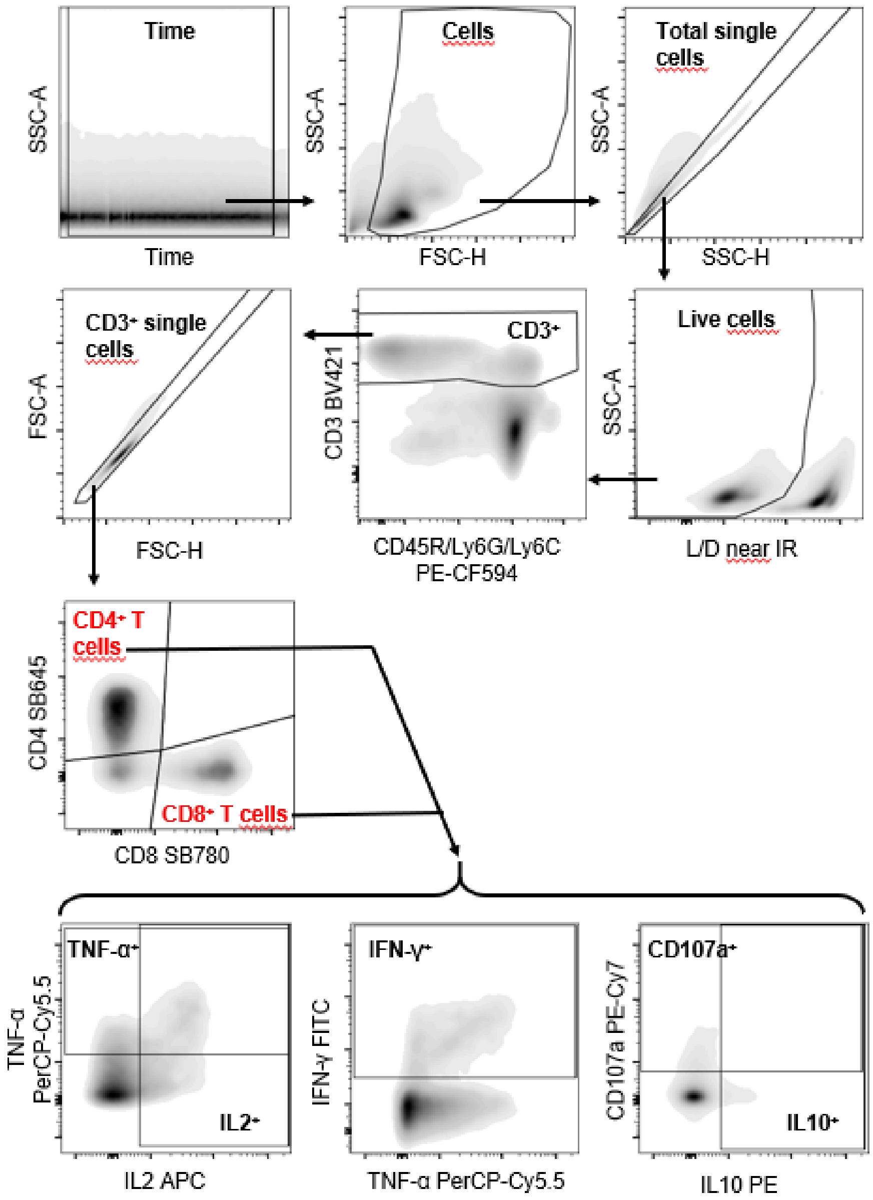

Figure S2. Flow cytometry gating strategy. At Days 10 and 28, splenocytes were stimulated with spike protein peptide pools and the functionality of T cells was measured by intracellular cytokine staining. After eliminating the doublets and dead cells, live CD3+ single cell population were divided into CD4+ and CD8+ T cell populations. For each population, the percentage of TNF-α+, IL2+, IFN-γ+, CD107a+ and IL10+ cells is presented.

Table S1. Listing of SARS-CoV-2 spike peptides.

Code Peptide	Pool	Molecular Weight Average	Sequence
1	1	1627.82	S-Q-C-V-N-L-T-T-R-T-Q-L-P-P-A
2	1	1839.89	D-L-F-L-P-F-F-S-N-V-T-W-F-H-A
3	1	1615.87	T-L-D-S-K-T-Q-S-L-L-I-V-N-N-A
4	1	1819.81	N-K-S-W-M-E-S-E-F-R-V-Y-S-S-A
5	1	1668.98	L-P-I-G-I-N-I-T-R-F-Q-T-L-L-A
6	1	1376.61	L-T-P-G-D-S-S-S-G-W-T-A-G-A-A
7	1	1765.89	S-V-Y-A-W-N-R-K-R-I-S-N-C-V-A
8	1	1674.86	A-D-S-F-V-I-R-G-D-E-V-R-Q-I-A

9	1	1581.84	D-E-V-R-Q-I-A-P-G-Q-T-G-K-I-A
10	1	1717.79	Y-N-Y-K-L-P-D-D-F-T-G-C-V-I-A
11	1	1808.92	L-K-P-F-E-R-D-I-S-T-E-I-Y-Q-A
12	1	1680.76	P-F-Q-Q-F-G-R-D-I-A-D-T-T-D-A
13	1	1619.78	A-V-L-Y-Q-D-V-N-C-T-E-V-P-V-A
14	1	1694.90	M-F-V-F-L-V-L-L-P-L-V-S-S-Q-C
15	1	1611.93	L-L-I-V-N-N-A-T-N-V-V-I-K-V-C
16	1	1654.74	L-K-Y-N-E-N-G-T-I-T-D-A-V-D-C
17	1	1715.78	Y-S-V-L-Y-N-S-A-S-F-S-T-F-K-C
18	1	1686.80	F-K-C-Y-G-V-S-P-T-K-L-N-D-L-C
19	1	1719.92	K-S-N-I-I-R-G-W-I-F-G-T-T-L-D
20	1	1830.00	F-K-I-Y-S-K-H-T-P-I-N-L-V-R-D
21	1	1783.90	R-T-F-L-L-K-Y-N-E-N-G-T-I-T-D
22	1	1681.82	G-Q-T-G-K-I-A-D-Y-N-Y-K-L-P-D
23	1	1968.06	Y-R-L-F-R-K-S-N-L-K-P-F-E-R-D
24	1	1808.95	K-K-F-L-P-F-Q-Q-F-G-R-D-I-A-D
25	1	1609.73	Q-D-V-N-C-T-E-V-P-V-A-I-H-A-D
26	1	1777.87	M-D-L-E-G-K-Q-G-N-F-K-N-L-R-E
27	1	1818.95	R-K-S-N-L-K-P-F-E-R-D-I-S-T-E
28	1	1643.79	I-A-D-T-T-D-A-V-R-D-P-Q-T-L-E
29	1	1681.75	S-N-Q-V-A-V-L-Y-Q-D-V-N-C-T-E
30	1	1792.87	T-N-S-F-T-R-G-V-Y-Y-P-D-K-V-F
31	1	1762.93	K-V-F-R-S-S-V-L-H-S-T-Q-D-L-F
32	1	1736.87	S-S-V-L-H-S-T-Q-D-L-F-L-P-F-F
33	1	1670.85	F-H-A-I-H-V-S-G-T-N-G-T-K-R-F
34	1	1724.85	N-N-A-T-N-V-V-I-K-V-C-E-F-Q-F
35	1	1692.70	S-S-A-N-N-C-T-F-E-Y-V-S-Q-P-F
36	1	1675.83	G-A-A-A-Y-Y-V-G-Y-L-Q-P-R-T-F
37	1	1714.80	T-N-L-C-P-F-G-E-V-F-N-A-T-R-F
38	1	1696.88	N-G-V-G-Y-Q-P-Y-R-V-V-V-L-S-F
39	1	1754.90	K-S-T-N-L-V-K-N-K-C-V-N-F-N-F
40	1	1550.82	G-L-T-G-T-G-V-L-T-E-S-N-K-K-F
41	1	1726.86	L-T-P-T-W-R-V-Y-S-T-G-S-N-V-F
42	1	1707.86	R-T-Q-L-P-P-A-Y-T-N-S-F-T-R-G
43	1	1638.78	N-V-T-W-F-H-A-I-H-V-S-G-T-N-G
44	1	1640.84	G-V-Y-F-A-S-T-E-K-S-N-I-I-R-G
45	1	1677.87	A-S-T-E-K-S-N-I-I-R-G-W-I-F-G
46	1	1839.95	N-F-K-N-L-R-E-F-V-F-K-N-I-D-G
47	1	1673.91	S-K-H-T-P-I-N-L-V-R-D-L-P-Q-G
48	1	1854.95	Y-L-Q-P-R-T-F-L-L-K-Y-N-E-N-G
49	1	1656.83	S-E-T-K-C-T-L-K-S-F-T-V-E-K-G
50	1	1676.86	S-I-V-R-F-P-N-I-T-N-L-C-P-F-G
51	1	1758.84	K-I-A-D-Y-N-Y-K-L-P-D-D-F-T-G
52	1	1572.78	V-I-A-W-N-S-N-N-L-D-S-K-V-G-G
53	1	1730.83	H-A-D-Q-L-T-P-T-W-R-V-Y-S-T-G
54	1	1857.97	V-F-K-N-I-D-G-Y-F-K-I-Y-S-K-H
55	1	1862.00	Y-Q-P-Y-R-V-V-V-L-S-F-E-L-L-H
56	1	1635.90	I-F-G-T-T-L-D-S-K-T-Q-S-L-L-I
57	1	1888.01	L-R-E-F-V-F-K-N-I-D-G-Y-F-K-I
58	1	1594.85	P-Q-G-F-S-A-L-E-P-L-V-D-L-P-I
59	1	1562.88	S-A-L-E-P-L-V-D-L-P-I-G-I-N-I
60	1	1697.87	T-D-A-V-R-D-P-Q-T-L-E-I-L-D-I
61	1	1876.85	F-Q-F-C-N-D-P-F-L-G-V-Y-Y-H-K
62	1	1750.86	Q-P-F-L-M-D-L-E-G-K-Q-G-N-F-K
63	1	1810.97	G-K-Q-G-N-F-K-N-L-R-E-F-V-F-K
64	1	1541.85	P-A-T-V-C-G-P-K-K-S-T-N-L-V-K

65	1	1805.79	N-C-T-F-E-Y-V-S-Q-P-F-L-M-D-L
66	1	1638.90	P-I-N-L-V-R-D-L-P-Q-G-F-S-A-L
67	1	1606.75	V-D-C-A-L-D-P-L-S-E-T-K-C-T-L
68	1	1727.91	Q-P-T-E-S-I-V-R-F-P-N-I-T-N-L
69	1	1663.82	S-F-S-T-F-K-C-Y-G-V-S-P-T-K-L
70	1	1773.89	L-D-S-K-V-G-G-N-Y-N-Y-L-Y-R-L
71	1	1512.80	V-I-T-P-G-T-N-T-S-N-Q-V-A-V-L
72	1	1718.86	T-N-G-T-K-R-F-D-N-P-V-L-P-F-N
73	1	1674.74	P-F-N-D-G-V-Y-F-A-S-T-E-K-S-N
74	1	1794.92	I-D-G-Y-F-K-I-Y-S-K-H-T-P-I-N
75	1	1802.93	N-F-R-V-Q-P-T-E-S-I-V-R-F-P-N
76	1	1710.80	F-P-N-I-T-N-L-C-P-F-G-E-V-F-N
77	1	1650.72	L-P-D-D-F-T-G-C-V-I-A-W-N-S-N
78	1	1542.63	Y-Q-A-G-S-T-P-C-N-G-V-E-G-F-N
79	1	1434.67	P-C-S-F-G-G-V-S-V-I-T-P-G-T-N
80	1	1731.82	P-P-A-Y-T-N-S-F-T-R-G-V-Y-Y-P
81	1	1753.81	N-V-V-I-K-V-C-E-F-Q-F-C-N-D-P
82	1	1815.00	R-F-Q-T-L-L-A-L-H-R-S-Y-L-T-P
83	1	1532.66	E-N-G-T-I-T-D-A-V-D-C-A-L-D-P
84	1	1974.07	Y-N-Y-L-Y-R-L-F-R-K-S-N-L-K-P
85	1	1665.77	E-R-D-I-S-T-E-I-Y-Q-A-G-S-T-P
86	1	1633.64	S-T-P-C-N-G-V-E-G-F-N-C-Y-F-P
87	1	1766.77	G-F-N-C-Y-F-P-L-Q-S-Y-G-F-Q-P
88	1	1641.73	Q-S-Y-G-F-Q-P-T-N-G-V-G-Y-Q-P
89	1	1553.78	L-S-F-E-L-L-H-A-P-A-T-V-C-G-P
90	1	1647.77	F-G-R-D-I-A-D-T-T-D-A-V-R-D-P
91	1	1592.78	C-T-E-V-P-V-A-I-H-A-D-Q-L-T-P
92	1	1645.84	P-L-V-S-S-Q-C-V-N-L-T-T-R-T-Q
93	1	1721.90	I-R-G-W-I-F-G-T-T-L-D-S-K-T-Q
94	1	1782.84	E-Y-V-S-Q-P-F-L-M-D-L-E-G-K-Q
95	1	1694.96	P-L-V-D-L-P-I-G-I-N-I-T-R-F-Q
96	1	1589.74	G-W-T-A-G-A-A-A-Y-Y-V-G-Y-L-Q
97	1	1707.91	T-G-V-L-T-E-S-N-K-K-F-L-P-F-Q
98	1	1430.69	G-G-V-S-V-I-T-P-G-T-N-T-S-N-Q
99	1	1775.87	S-F-T-V-E-K-G-I-Y-Q-T-S-N-F-R
100	1	1800.90	V-F-N-A-T-R-F-A-S-V-Y-A-W-N-R
101	1	1761.83	D-L-C-F-T-N-V-Y-A-D-S-F-V-I-R
102	1	1825.94	T-E-S-N-K-K-F-L-P-F-Q-Q-F-G-R
103	1	1675.84	N-L-T-T-R-T-Q-L-P-P-A-Y-T-N-S
104	1	1787.87	P-F-F-S-N-V-T-W-F-H-A-I-H-V-S
105	1	1794.86	N-D-P-F-L-G-V-Y-Y-H-K-N-N-K-S
106	1	1854.86	L-G-V-Y-Y-H-K-N-N-K-S-W-M-E-S
107	1	1782.02	I-N-I-T-R-F-Q-T-L-L-A-L-H-R-S
108	1	1628.84	L-L-A-L-H-R-S-Y-L-T-P-G-D-S-S
109	1	1853.99	T-R-F-A-S-V-Y-A-W-N-R-K-R-I-S
110	1	1702.79	R-I-S-N-C-V-A-D-Y-S-V-L-Y-N-S
111	1	1624.70	C-V-A-D-Y-S-V-L-Y-N-S-A-S-F-S
112	1	1659.71	Y-N-S-A-S-F-S-T-F-K-C-Y-G-V-S
113	1	1702.78	T-K-L-N-D-L-C-F-T-N-V-Y-A-D-S
114	1	1639.72	F-T-G-C-V-I-A-W-N-S-N-N-L-D-S
115	1	1848.95	V-G-G-N-Y-N-Y-L-Y-R-L-F-R-K-S
116	1	1555.74	F-N-F-N-G-L-T-G-T-G-V-L-T-E-S
117	1	1699.83	R-D-P-Q-T-L-E-I-L-D-I-T-P-C-S
118	1	1597.90	L-V-L-L-P-L-V-S-S-Q-C-V-N-L-T
119	1	1797.89	Y-Y-P-D-K-V-F-R-S-S-V-L-H-S-T
120	1	1751.84	H-S-T-Q-D-L-F-L-P-F-F-S-N-V-T

121	1	1639.78	N-P-V-L-P-F-N-D-G-V-Y-F-A-S-T
122	1	1736.70	M-E-S-E-F-R-V-Y-S-S-A-N-N-C-T
123	1	1649.73	H-R-S-Y-L-T-P-G-D-S-S-S-G-W-T
124	1	1561.71	I-T-D-A-V-D-C-A-L-D-P-L-S-E-T
125	1	1681.85	L-D-P-L-S-E-T-K-C-T-L-K-S-F-T
126	1	1716.87	C-T-L-K-S-F-T-V-E-K-G-I-Y-Q-T
127	1	1766.88	E-K-G-I-Y-Q-T-S-N-F-R-V-Q-P-T
128	1	1637.87	V-I-R-G-D-E-V-R-Q-I-A-P-G-Q-T
129	1	1650.94	R-V-V-V-L-S-F-E-L-L-H-A-P-A-T
130	1	1521.82	L-L-H-A-P-A-T-V-C-G-P-K-K-S-T
131	1	1709.88	L-V-K-N-K-C-V-N-F-N-F-N-G-L-T
132	1	1507.75	L-D-I-T-P-C-S-F-G-G-V-S-V-I-T
133	1	1772.91	T-R-G-V-Y-Y-P-D-K-V-F-R-S-S-V
134	1	1627.80	H-V-S-G-T-N-G-T-K-R-F-D-N-P-V
135	1	1612.90	K-T-Q-S-L-L-I-V-N-N-A-T-N-V-V
136	1	1744.78	K-V-C-E-F-Q-F-C-N-D-P-F-L-G-V
137	1	1953.91	Y-H-K-N-N-K-S-W-M-E-S-E-F-R-V
138	1	1798.79	F-R-V-Y-S-S-A-N-N-C-T-F-E-Y-V
139	1	1639.88	V-R-D-L-P-Q-G-F-S-A-L-E-P-L-V
140	1	1504.64	D-S-S-S-G-W-T-A-G-A-A-A-Y-Y-V
141	1	1767.87	Y-Q-T-S-N-F-R-V-Q-P-T-E-S-I-V
142	1	1809.88	W-N-R-K-R-I-S-N-C-V-A-D-Y-S-V
143	1	1606.79	G-V-S-P-T-K-L-N-D-L-C-F-T-N-V
144	1	1683.80	T-N-V-Y-A-D-S-F-V-I-R-G-D-E-V
145	1	1525.66	S-T-E-I-Y-Q-A-G-S-T-P-C-N-G-V
146	1	1716.80	Y-F-P-L-Q-S-Y-G-F-Q-P-T-N-G-V
147	1	1723.86	F-Q-P-T-N-G-V-G-Y-Q-P-Y-R-V-V
148	1	1617.86	C-G-P-K-K-S-T-N-L-V-K-N-K-C-V
149	1	1569.75	K-C-V-N-F-N-F-N-G-L-T-G-T-G-V
150	1	1563.77	T-L-E-I-L-D-I-T-P-C-S-F-G-G-V
151	1	1607.77	G-T-N-T-S-N-Q-V-A-V-L-Y-Q-D-V
152	1	1702.90	P-V-A-I-H-A-D-Q-L-T-P-T-W-R-V
153	1	1779.88	K-R-F-D-N-P-V-L-P-F-N-D-G-V-Y
154	1	1923.02	Y-Y-V-G-Y-L-Q-P-R-T-F-L-L-K-Y
155	1	1703.82	P-F-G-E-V-F-N-A-T-R-F-A-S-V-Y
156	1	1637.79	Q-I-A-P-G-Q-T-G-K-I-A-D-Y-N-Y
157	1	1657.72	N-S-N-N-L-D-S-K-V-G-G-N-Y-N-Y
158	1	1736.74	N-G-V-E-G-F-N-C-Y-F-P-L-Q-S-Y
159	2	1770.87	W-R-V-Y-S-T-G-S-N-V-F-Q-T-R-A
160	2	1550.78	G-S-F-C-T-Q-L-N-R-A-L-T-G-I-A
161	2	1647.80	G-I-A-V-E-Q-D-K-N-T-Q-E-V-F-A
162	2	1707.90	F-I-E-D-L-L-F-N-K-V-T-L-A-D-A
163	2	1581.86	G-L-T-V-L-P-P-L-L-T-D-E-M-I-A
164	2	1638.81	L-T-D-E-M-I-A-Q-Y-T-S-A-L-L-A
165	2	1505.76	G-W-T-F-G-A-G-A-A-L-Q-I-P-F-A
166	2	1475.72	G-A-G-A-A-L-Q-I-P-F-A-M-Q-M-A
167	2	1760.97	L-Q-S-L-Q-T-Y-V-T-Q-Q-L-I-R-A
168	2	1572.76	K-N-F-T-T-A-P-A-I-C-H-D-G-K-A
169	2	1567.65	N-N-S-Y-E-C-D-I-P-I-G-A-G-I-C
170	2	1680.71	V-S-M-T-K-T-S-V-D-C-T-M-Y-I-C
171	2	1596.72	Y-G-D-C-L-G-D-I-A-A-R-D-L-I-C
172	2	1700.76	M-S-E-C-V-L-G-Q-S-K-R-V-D-F-C
173	2	1559.87	I-A-G-L-I-A-I-V-M-V-T-I-M-L-C
174	2	1627.56	M-V-T-I-M-L-C-C-M-T-S-C-C-S-C
175	2	1584.53	M-L-C-C-M-T-S-C-C-S-C-L-K-G-C
176	2	1665.67	C-L-I-G-A-E-H-V-N-N-S-Y-E-C-D

177	2	1627.84	T-Q-L-N-R-A-L-T-G-I-A-V-E-Q-D
178	2	1624.84	K-V-T-L-A-D-A-G-F-I-K-Q-Y-G-D
179	2	1571.68	A-D-A-G-F-I-K-Q-Y-G-D-C-L-G-D
180	2	1654.92	Q-K-F-N-G-L-T-V-L-P-P-L-L-T-D
181	2	1550.75	Q-L-S-S-N-F-G-A-I-S-S-V-L-N-D
182	2	1626.71	Q-I-I-T-T-D-N-T-F-V-S-G-N-C-D
183	2	1636.77	N-C-D-V-V-I-G-I-V-N-N-T-V-Y-D
184	2	1849.87	K-E-E-L-D-K-Y-F-K-N-H-T-S-P-D
185	2	1726.91	R-L-N-E-V-A-K-N-L-N-E-S-L-I-D
186	2	1583.51	K-G-C-C-S-C-G-S-C-C-K-F-D-E-D
187	2	1712.94	I-L-P-D-P-S-K-P-S-K-R-S-F-I-E
188	2	1590.79	E-I-R-A-S-A-N-L-A-A-T-K-M-S-E
189	2	1884.84	S-N-G-T-H-W-F-V-T-Q-R-N-F-Y-E
190	2	1807.84	V-Y-D-P-L-Q-P-E-L-D-S-F-K-E-E
191	2	1783.93	K-E-I-D-R-L-N-E-V-A-K-N-L-N-E
192	2	1762.89	L-N-E-S-L-I-D-L-Q-E-L-G-K-Y-E
193	2	1675.73	D-S-T-E-C-S-N-L-L-L-Q-Y-G-S-F
194	2	1762.95	P-S-K-P-S-K-R-S-F-I-E-D-L-L-F
195	2	1632.86	L-G-D-I-A-A-R-D-L-I-C-A-Q-K-F
196	2	1820.93	Q-N-V-L-Y-E-N-Q-K-L-I-A-N-Q-F
197	2	1790.92	V-F-L-H-V-T-Y-V-P-A-Q-E-K-N-F
198	2	1711.81	I-C-H-D-G-K-A-H-F-P-R-E-G-V-F
199	2	1778.84	F-P-R-E-G-V-F-V-S-N-G-T-H-W-F
200	2	1659.71	A-E-H-V-N-N-S-Y-E-C-D-I-P-I-G
201	2	1689.93	V-K-Q-I-Y-K-T-P-P-I-K-D-F-G-G
202	2	1450.74	L-L-A-G-T-I-T-S-G-W-T-F-G-A-G
203	2	1618.86	Q-A-L-N-T-L-V-K-Q-L-S-S-N-F-G
204	2	1684.89	D-K-V-E-A-E-V-Q-I-D-R-L-I-T-G
205	2	1493.71	S-A-N-L-A-A-T-K-M-S-E-C-V-L-G
206	2	1655.78	K-G-Y-H-L-M-S-F-P-Q-S-A-P-H-G
207	2	1600.80	G-K-A-H-F-P-R-E-G-V-F-V-S-N-G
208	2	1539.68	T-D-N-T-F-V-S-G-N-C-D-V-V-I-G
209	2	1734.81	D-K-Y-F-K-N-H-T-S-P-D-V-D-L-G
210	2	1553.72	K-N-H-T-S-P-D-V-D-L-G-D-I-S-G
211	2	2072.05	K-Y-E-Q-Y-I-K-W-P-W-Y-I-W-L-G
212	2	1912.00	Y-I-K-W-P-W-Y-I-W-L-G-F-I-A-G
213	2	1484.46	M-T-S-C-C-S-C-L-K-G-C-C-S-C-G
214	2	1683.70	C-C-K-F-D-E-D-D-S-E-P-V-L-K-G
215	2	1614.78	N-V-F-Q-T-R-A-G-C-L-I-G-A-E-H
216	2	1626.77	P-A-Q-E-K-N-F-T-T-A-P-A-I-C-H
217	2	1911.93	L-D-S-F-K-E-E-L-D-K-Y-F-K-N-H
218	2	1679.83	D-E-D-D-S-E-P-V-L-K-G-V-K-L-H
219	2	1552.76	S-T-G-S-N-V-F-Q-T-R-A-G-C-L-I
220	2	1730.95	I-G-V-T-Q-N-V-L-Y-E-N-Q-K-L-I
221	2	1751.87	Y-E-N-Q-K-L-I-A-N-Q-F-N-S-A-I
222	2	1976.98	H-W-F-V-T-Q-R-N-F-Y-E-P-Q-I-I
223	2	1485.76	D-L-G-D-I-S-G-I-N-A-S-V-V-N-I
224	2	1583.88	I-S-G-I-N-A-S-V-V-N-I-Q-K-E-I
225	2	1731.96	P-W-Y-I-W-L-G-F-I-A-G-L-I-A-I
226	2	1759.03	V-F-A-Q-V-K-Q-I-Y-K-T-P-P-I-K
227	2	1652.81	F-G-G-F-N-F-S-Q-I-L-P-D-P-S-K
228	2	1648.94	L-L-F-N-K-V-T-L-A-D-A-G-F-I-K
229	2	1504.80	K-I-Q-D-S-L-S-S-T-A-S-A-L-G-K
230	2	1767.97	V-N-I-Q-K-E-I-D-R-L-N-E-V-A-K
231	2	1851.99	L-I-D-L-Q-E-L-G-K-Y-E-Q-Y-I-K
232	2	1483.48	C-S-C-L-K-G-C-C-S-C-G-S-C-C-K

233	2	1625.83	R-S-V-A-S-Q-S-I-I-A-Y-T-M-S-L
234	2	1618.87	A-I-P-T-N-F-T-I-S-V-T-T-E-I-L
235	2	1688.78	C-S-N-L-L-L-Q-Y-G-S-F-C-T-Q-L
236	2	1725.88	L-L-Q-Y-G-S-F-C-T-Q-L-N-R-A-L
237	2	1609.87	L-G-K-L-Q-D-V-V-N-Q-N-A-Q-A-L
238	2	1628.83	L-M-S-F-P-Q-S-A-P-H-G-V-V-F-L
239	2	1697.91	V-A-K-N-L-N-E-S-L-I-D-L-Q-E-L
240	2	1650.84	N-F-T-I-S-V-T-T-E-I-L-P-V-S-M
241	2	1522.70	P-I-G-A-G-I-C-A-S-Y-Q-T-Q-T-N
242	2	1583.74	S-Q-S-I-I-A-Y-T-M-S-L-G-A-E-N
243	2	1640.54	D-C-T-M-Y-I-C-G-D-S-T-E-C-S-N
244	2	1799.87	A-L-Q-I-P-F-A-M-Q-M-A-Y-R-F-N
245	2	1722.86	R-F-N-G-I-G-V-T-Q-N-V-L-Y-E-N
246	2	1556.81	T-A-S-A-L-G-K-L-Q-D-V-V-N-Q-N
247	2	1640.89	T-Q-Q-L-I-R-A-A-E-I-R-A-S-A-N
248	2	1838.87	T-Q-R-N-F-Y-E-P-Q-I-I-T-T-D-N
249	2	1548.75	F-V-S-G-N-C-D-V-V-I-G-I-V-N-N
250	2	1711.91	N-A-S-V-V-N-I-Q-K-E-I-D-R-L-N
251	2	1548.73	A-E-N-S-V-A-Y-S-N-N-S-I-A-I-P
252	2	1678.86	P-I-K-D-F-G-G-F-N-F-S-Q-I-L-P
253	2	1612.89	L-I-C-A-Q-K-F-N-G-L-T-V-L-P-P
254	2	1664.83	V-T-Y-V-P-A-Q-E-K-N-F-T-T-A-P
255	2	1640.87	V-I-G-I-V-N-N-T-V-Y-D-P-L-Q-P
256	2	1620.53	S-C-G-S-C-C-K-F-D-E-D-D-S-E-P
257	2	1538.66	E-C-D-I-P-I-G-A-G-I-C-A-S-Y-Q
258	2	1657.72	Y-I-C-G-D-S-T-E-C-S-N-L-L-L-Q
259	2	1642.85	R-A-L-T-G-I-A-V-E-Q-D-K-N-T-Q
260	2	1790.87	E-Q-D-K-N-T-Q-E-V-F-A-Q-V-K-Q
261	2	1479.73	T-I-T-S-G-W-T-F-G-A-G-A-A-L-Q
262	2	1643.93	K-L-I-A-N-Q-F-N-S-A-I-G-K-I-Q
263	2	1734.92	I-T-G-R-L-Q-S-L-Q-T-Y-V-T-Q-Q
264	2	1662.88	P-H-G-V-V-F-L-H-V-T-Y-V-P-A-Q
265	2	1680.79	G-I-C-A-S-Y-Q-T-Q-T-N-S-P-R-R
266	2	1712.91	N-F-S-Q-I-L-P-D-P-S-K-P-S-K-R
267	2	1668.82	F-I-K-Q-Y-G-D-C-L-G-D-I-A-A-R
268	2	1604.84	N-F-G-A-I-S-S-V-L-N-D-I-L-S-R
269	2	1769.95	L-S-R-L-D-K-V-E-A-E-V-Q-I-D-R
270	2	1788.97	Q-T-Y-V-T-Q-Q-L-I-R-A-A-E-I-R
271	2	1607.80	A-A-T-K-M-S-E-C-V-L-G-Q-S-K-R
272	2	1619.79	T-A-P-A-I-C-H-D-G-K-A-H-F-P-R
273	2	1733.85	G-V-F-V-S-N-G-T-H-W-F-V-T-Q-R
274	2	1540.73	T-R-A-G-C-L-I-G-A-E-H-V-N-N-S
275	2	1643.83	Q-T-N-S-P-R-R-A-R-S-V-A-S-Q-S
276	2	1542.65	M-S-L-G-A-E-N-S-V-A-Y-S-N-N-S
277	2	1592.82	S-V-T-T-E-I-L-P-V-S-M-T-K-T-S
278	2	1716.84	Y-K-T-P-P-I-K-D-F-G-G-F-N-F-S
279	2	1690.84	L-P-P-L-L-T-D-E-M-I-A-Q-Y-T-S
280	2	1620.80	N-Q-F-N-S-A-I-G-K-I-Q-D-S-L-S
281	2	1463.74	S-A-I-G-K-I-Q-D-S-L-S-S-T-A-S
282	2	1640.87	N-Q-N-A-Q-A-L-N-T-L-V-K-Q-L-S
283	2	1550.82	T-L-V-K-Q-L-S-S-N-F-G-A-I-S-S
284	2	1697.93	A-E-V-Q-I-D-R-L-I-T-G-R-L-Q-S
285	2	1726.82	S-K-R-V-D-F-C-G-K-G-Y-H-L-M-S
286	2	1715.73	D-F-C-G-K-G-Y-H-L-M-S-F-P-Q-S
287	2	1773.84	F-Y-E-P-Q-I-I-T-T-D-N-T-F-V-S
288	2	1702.79	V-N-N-T-V-Y-D-P-L-Q-P-E-L-D-S

289	2	1458.67	S-P-D-V-D-L-G-D-I-S-G-I-N-A-S
290	2	1627.78	I-A-I-V-M-V-T-I-M-L-C-C-M-T-S
291	2	1610.78	V-A-Y-S-N-N-S-I-A-I-P-T-N-F-T
292	2	1590.81	N-N-S-I-A-I-P-T-N-F-T-I-S-V-T
293	2	1622.78	E-I-L-P-V-S-M-T-K-T-S-V-D-C-T
294	2	1622.65	K-T-S-V-D-C-T-M-Y-I-C-G-D-S-T
295	2	1795.94	N-T-Q-E-V-F-A-Q-V-K-Q-I-Y-K-T
296	2	1795.97	S-K-R-S-F-I-E-D-L-L-F-N-K-V-T
297	2	1619.83	A-A-R-D-L-I-C-A-Q-K-F-N-G-L-T
298	2	1552.81	M-I-A-Q-Y-T-S-A-L-L-A-G-T-I-T
299	2	1540.77	Y-T-S-A-L-L-A-G-T-I-T-S-G-W-T
300	2	1526.84	I-R-A-A-E-I-R-A-S-A-N-L-A-A-T
301	2	1602.91	W-L-G-F-I-A-G-L-I-A-I-V-M-V-T
302	2	1699.87	D-D-S-E-P-V-L-K-G-V-K-L-H-Y-T
303	2	1749.88	S-Y-Q-T-Q-T-N-S-P-R-R-A-R-S-V
304	2	1700.81	P-F-A-M-Q-M-A-Y-R-F-N-G-I-G-V
305	2	1696.82	Q-M-A-Y-R-F-N-G-I-G-V-T-Q-N-V
306	2	1475.77	S-L-S-S-T-A-S-A-L-G-K-L-Q-D-V
307	2	1625.83	Q-D-V-V-N-Q-N-A-Q-A-L-N-T-L-V
308	2	1670.95	I-S-S-V-L-N-D-I-L-S-R-L-D-K-V
309	2	1712.92	L-N-D-I-L-S-R-L-D-K-V-E-A-E-V
310	2	1673.92	P-R-R-A-R-S-V-A-S-Q-S-I-I-A-Y
311	2	1588.73	I-A-Y-T-M-S-L-G-A-E-N-S-V-A-Y
312	2	1775.98	I-D-R-L-I-T-G-R-L-Q-S-L-Q-T-Y
313	2	1655.83	V-L-G-Q-S-K-R-V-D-F-C-G-K-G-Y
314	2	1650.84	P-Q-S-A-P-H-G-V-V-F-L-H-V-T-Y
315	2	1852.90	L-Q-P-E-L-D-S-F-K-E-E-L-D-K-Y
316	2	2029.98	Q-E-L-G-K-Y-E-Q-Y-I-K-W-P-W-Y

Table S2. Polyfunctionality of CD8+ T Cells.

	**DNA Spike**				**DNA Spike + rec muGM-CSF**				**DNA Spike + Capsule muGM-CSF**			
	**Neg**	**Pool 1**	**Pool 2**	**Pos**	**Neg**	**Pool 1**	**Pool 2**	**Pos**	Neg	Pool 1	Pool 2	Pos
CD107a+ IL10+ TNFα+ IL2+ IFNγ+	0	0	0	0.0231	0	0	0	0	0	0.049	0	0
CD107a+ IL10- TNFα+ IL2+ IFNγ+	0	0.0935	0	0.8062	0	0.1144	0.0571	0.6899	0	0.3814	0.0974	0.7989
CD107a+ IL10+ TNFα+ IL2- IFNγ+	0	0.0935	0.0576	0.0037	0	0	0	0.0355	0	0.0844	0.0913	0.0239
CD107a+ IL10+ TNFα- IL2+ IFNγ+	0	0	0	0.013	0.0513	0	0	0	0	0	0	0
CD107a+ IL10+ TNFα+ IL2+ IFNγ-	0	0	0	0.0047	0	0	0	0	0	0	0	0
CD107a- IL10+ TNFα+ IL2+ IFNγ+	0	0.1085	0.0614	0.0751	0.4649	0.0539	1.2603	0.0431	0.4898	0.4958	0.0974	0.0522
CD107a+ IL10- TNFα+ IL2- IFNγ+	0.1348	0.4341	0.119	3.258	0.0664	0.8853	0.4188	2.9234	0.1111	1.2613	0.9644	2.4974
CD107a+ IL10+ TNFα- IL2- IFNγ+	0	0.0452	0.119	0.0366	0	0	0.1257	0.0634	0	0.0381	0.0882	0.0837
CD107a+ IL10- TNFα- IL2+ IFNγ+	0	0	0	0.742	0	0	0	0.381	0	0	0	0.6271
CD107a+ IL10+ TNFα- IL2+ IFNγ-	0	0	0	0	0	0	0	0	0	0	0	0
CD107a+ IL10+ TNFα+ IL2- IFNγ-	0.149	0.0452	0.0537	0	0	0.0471	0.2513	0	0	0	0	0.0044

CD107a- IL10+ TNFα+ IL2+ IFNγ-	0	0	0	0.0039	0	0	0	0.0098	0	0	0	0.0031
CD107a- IL10+ TNFα- IL2+ IFNγ+	0.0603	0.1869	0.1727	0.0229	0.1268	0.2222	0.3617	0.0244	0.1969	0.4222	0.0913	0.0542
CD107a+ IL10- TNFα+ IL2+ IFNγ-	0	0.1568	0.0614	0.0506	0	0.101	0.1599	0.0358	0	0.2506	0	0.2222
CD107a- IL10- TNFα+ IL2+ IFNγ+	0	0.1869	0.1765	3.9703	0	0.2727	0.0685	4.7895	0	0.2098	0.1856	4.3106
CD107a- IL10+ TNFα+ IL2- IFNγ+	0	0.4492	0.2878	0.0835	0	0.4376	0.3808	0.0191	0.1187	0.1389	0.1947	0.0697
CD107a+ IL10- TNFα- IL2- IFNγ+	0.653	0.6693	1.3049	13.6859	0.818	0.717	1.7134	10.7549	0.871	1.6127	1.4695	11.2435
CD107a+ IL10- TNFα+ IL2- IFNγ-	0.2094	0.9647	1.0209	0.1673	0	0.8112	1.2641	0.1543	0.3434	1.599	1.798	0.0918
CD107a+ IL10- TNFα- IL2+ IFNγ-	0	0	0	0.0806	0	0	0.0419	0.0366	0.0429	0.0409	0.1339	0.1275
CD107a+ IL10+ TNFα- IL2- IFNγ-	0.5217	0.2321	0.1765	0.0051	0.166	0.1683	0.4683	0.0151	0.0631	0.1743	0.1856	0.0075
CD107a- IL10- TNFα+ IL2- IFNγ+	0.0745	1.6159	1.397	16.8726	0.1207	2.5481	1.7972	17.355	0.048	1.8088	1.5729	11.7704
CD107a- IL10- TNFα- IL2+ IFNγ+	0	0	0	3.5018	0	0	0.0685	3.0871	0	0.049	0	4.1573
CD107a- IL10+ TNFα- IL2- IFNγ+	0.2129	0.5457	0.7714	0.137	0.1751	0.1919	0.7958	0.1825	0.4898	0.4222	0.1734	0.1766
CD107a- IL10- TNFα+ IL2+ IFNγ-	0	0.4884	0.2917	0.3092	0	0.3669	0.1637	0.3428	0	0.5748	0.3134	1.019
CD107a- IL10+ TNFα+ IL2- IFNγ-	0	0.5246	0.1228	0	0.0513	0.2255	0.2361	0	0	0.1335	0	0
CD107a- IL10+ TNFα- IL2+ IFNγ-	0.1207	0	0	0	0.1721	0	0.1371	0	0.0631	0	0.0974	0.004
CD107a+ IL10- TNFα- IL2- IFNγ-	76.81	46.9919	57.2407	5.776	79.942	50.0648	54.1038	4.4619	81.8791	52.0579	59.3572	3.4489
CD107a- IL10- TNFα- IL2- IFNγ+	1.4872	7.2737	7.8387	48.873	3.7216	13.098	10.731	52.9218	5.2914	11.2846	10.9686	57.5229
CD107a- IL10- TNFα+ IL2- IFNγ-	3.9263	17.1036	14.1057	0.825	4.3977	19.7298	14.4932	1.0738	4.6774	21.09	17.4611	0.7123
CD107a- IL10- TNFα- IL2+ IFNγ-	4.5859	3.5361	4.0325	0.642	5.851	3.2414	5.9868	0.5798	3.9039	3.1922	3.9856	0.947
CD107a- IL10+ TNFα- IL2- IFNγ-	11.0597	18.2354	10.5689	0.0311	3.865	5.1252	4.8981	0.0195	1.4126	2.6143	0.6725	0.0238
